# Frailty as a predictor of mortality: a comparative cohort study of older adults in Costa Rica and the United States

**DOI:** 10.1186/s12889-023-16900-4

**Published:** 2023-10-10

**Authors:** Carolina Santamaría-Ulloa, Amanda J. Lehning, Mónica V. Cortés-Ortiz, Ericka Méndez-Chacón

**Affiliations:** 1 Instituto de Investigaciones en Salud, Universidad de Costa Rica, San José, Costa Rica; 2 School of Social Work, University of Maryland Baltimore, Maryland, United States of America; 3 Graduate School Student Fellow, University of Maryland Baltimore, Maryland, United States of America; 4 Escuela de Estadística, Universidad de Costa Rica, San José, Costa Rica

**Keywords:** Aging, Mortality, Frailty, CRELES, NHATS

## Abstract

**Background:**

Frailty is a common condition among older adults that results from aging-related declines in multiple systems. Frailty increases older adults’ vulnerability to negative health outcomes, including loss of mobility, falls, hospitalizations, and mortality. The aim of this study is to examine the association between frailty and mortality in older adults from Costa Rica and the United States.

**Methods:**

This prospective cohort study uses secondary nationally-representative data of community-dwelling older adults from the Costa Rican Longevity and Healthy Aging Study (CRELES, n = 1,790) and the National Health & Aging Trends Study (NHATS, n = 6,680). Frailty status was assessed using Physical Frailty Phenotype, which includes the following five criteria: shrinking, exhaustion, low physical activity, muscle weakness, and slow gait. We used Cox proportional hazard models to examine the association between frailty and all-cause mortality, including sociodemographic characteristics and health behaviors as covariates in the models. Mortality follow-up time was right censored at 8 years from the date at baseline interview.

**Results:**

The death hazard for frail compared to non-frail older adults was three-fold in Costa Rica (HR = 3.14, 95% CI: 2.13–4.62) and four-fold in the White US (HR = 4.02, 95% CI: 3.04–5.32). Older age, being male, and smoking increased mortality risk in both countries. High education was a protective factor in the US, whereas being married/in union was a protective factor in Costa Rica. In the US, White older adults had a lower risk of death compared to all other races and ethnicities.

**Conclusions:**

Results indicate that frailty can have a differential impact on mortality depending on the country. Access to universal health care across the life course in Costa Rica and higher levels of stress and social isolation in the US may explain differences observed in end-of-life trajectories among frail older adults.

**Supplementary Information:**

The online version contains supplementary material available at 10.1186/s12889-023-16900-4.

## Background

Frailty is a syndrome that results from aging-related declines in multiple systems and leads to an increased vulnerability to adverse health outcomes [1]. Because of its age-dependent nature, frailty is common in older adults [2, 3]. A systematic review and meta-analysis estimated an incidence rate of frailty of 40 cases per 1000 person-years in community-dwelling older adults 60 years or older from developed countries [4]. From a biological standpoint, this syndrome results from decreased reserves in multiple physiological systems, which leads to loss of homeostatic capability to cope with stressors. From a clinical standpoint, this condition becomes clinically visible above a threshold of severity [5]. Adverse health outcomes associated with frailty include loss of mobility, falls, hospitalization, and mortality. Furthermore, this syndrome also poses challenges for families, caregivers, and social support institutions [6].

Several scales have been developed to operationalize the measurement of frailty in older adults [7]. In 2001, Fried and colleagues proposed their landmark frailty phenotype measurement called Fried’s Physical Frailty Phenotype (PFP), which has become the most widely used frailty screening tool in population studies. The PFP scale consists of five components: (1) shrinking, (2) exhaustion, (3) low physical activity level, (4) muscle weakness, and (5) slow gait [8]. These five frailty-identifying characteristics measure the negative energy balance, sarcopenia, and diminished strength and tolerance for exertion resulting from multiple systems decline [5].

Frailty impacts not only individuals but also their families and communities. For example, there is emerging evidence that frailty is associated with increased risk of physical and psychological burden among family caregivers [9]. Furthermore, because older adults living with frailty require a great deal of care, it poses a financial burden on health care systems. Population studies are a valuable tool to determine prevalence and to project the impact this syndrome has on aging societies. Frailty has been shown to be a strong predictor of mortality in previous systematic reviews and meta-analyses in both developed and developing countries [10, 11]. Most research on the association between frailty and mortality has focused on developed countries, however this is also an important issue in developing nations where population aging has occurred at a faster pace and is challenging health systems. Costa Rica is a Central American developing country that has achieved outstanding health standards. Total life expectancy in Costa Rica is 81 years, higher than the 76 years life expectancy in the United States (US). This is despite Costa Rica having a per capita gross national income (GNI) of less than one-third that of the US [12]. Costa Rica’s high health standards have been attributed to their universal health care system with a strong focus in the delivery of primary care [13] particularly to remote and poor populations [14], in contrast to the low financial access and fragmented health care in the US. Indeed, Costa Rica abolished its military in 1949, reallocating this funding to health and education [15]. The share of public health expenditures in Costa Rica is similar to Canada and considerably higher than the US [16]. Despite these documented differences, few studies compare the experience of aging in these two countries.

The aim of this study is to compare the association between frailty and mortality in older adults from Costa Rica, a developing country, and the US, a developed country. Although previous studies have demonstrated that frailty increases the risk of death in different populations, we hypothesize that frailty can have a differential impact on mortality depending on the country, which may be due to differences in the social and economic contexts. Results from this study can inform public policies aimed at reducing the risk factors for frailty and the risk of death due to frailty in older adult populations.

## Methods

### Study design

This is a prospective cohort study, using secondary nationally representative data from community-dwelling participants enrolled in the Costa Rican Longevity and Healthy Aging Study (CRELES, for its Spanish acronym) and the National Health & Aging Trends Study (NHATS).

The Costa Rican cohort (CRELES) is a longitudinal study based on a national sample representing older adults in Costa Rica. In the first stage, a random selection was drawn from the census of 2000, totaling 9,600 individuals 55 years of age or older, with an oversampling of the oldest old. In the second stage, a sub-sampling consisting of 60 Health Areas was selected; Health Areas are the administrative division units that are used for healthcare providing purposes. The sample covers 59% of the national territory. The sub-sampling for the longitudinal study originally included around 5,000 individuals from the census of 2000; of those, it was possible to locate and interview 2,827 out of 3,024 eligible individuals ages 60 and older, which resulted in a response rate of 93% [17]. Data collection consisted of in-person interviews and biomarker collection in the participant’s home conducted by professionally trained interviewers.

The US cohort (NHATS) is a panel study based on a national sample representing older adults receiving Medicare. The NHATS sample was age-stratified, with individuals selected from 5-year age groups between the ages of 65 and 90, and from individuals ages 90 and older. The study oversampled individuals at the oldest age group and Black older adults. Data collection consisted of in-person interviews in the participant’s home conducted by professionally trained interviewers. The first wave in 2011 contained data on 8,245 older adults and had a response rate of 71%.

This current study was restricted to community-dwelling older adults who were able to make self-reports without the need of a proxy respondent and who were ages 65+. It is restricted to community-dwelling respondents because frailty is expected to be more prevalent in non-community settings. It is restricted to participants who did not need a proxy respondent because exhaustion and unintentional weight loss, two measurements that are part of the frailty index, may be unreliable if based on a proxy report. Although CRELES interviewed participants starting at the age of 60, only participants 65 + were included for this study in order to make both cohorts comparable in terms of age.

From the initial cohort size for Costa Rica (n = 2,827), 334 participants were excluded because they were aged 60 to 64, and 703 participants were excluded because they were unable to complete the survey themselves and needed a proxy respondent. The resulting sample size in this study was n = 1,790 for CRELES.

From the initial cohort size for the United States (n = 8,245), 1,048 participants were excluded because they were living in a nursing home or a long-term care institution, and 517 participants were excluded because they were unable to complete the survey themselves and needed a proxy respondent. The resulting sample size used in this study was n = 6,680 for NHATS.

### Physical frailty phenotype assessment

Frailty status was assessed by the five binary criteria of the PFP [8]: shrinking, exhaustion, low physical activity, muscle weakness, and slow gait. As some specific questions and measurements collected for CRELES and NHATS differed from each other and from the study on which the phenotype is based [8], we adapted the criteria definitions to make them comparable using the available data in both cohorts. Three out of five components of the PFP (shrinking, exhaustion, and low physical activity) were self-reports, whereas the other two (muscle weakness and slow gait) were objective measures of tasks conducted during interviews.

Shrinking was defined as self-report of unintentional weight loss of 5 + kg (11 + pounds) in the last 6 months for CRELES participants, or unintentional weight loss of 4.5 + kg (10 + pounds) in the last 12 months for NHATS participants. Exhaustion was defined as self-report of daily severe fatigue or exhaustion over the last year for CRELES participants, or low energy or exhaustion that limited their activities in the last month for NHATS participants. For CRELES participants, low physical activity was defined as not exercising regularly or engaging in vigorous physical activities (such as playing a sport, jogging, dancing or doing heavy work) at least three times a week during the last year. For NHATS participants, low physical activity was defined as not spending time in vigorous physical activities, such as working out, swimming, running, biking or playing a sport, in the last month.

Muscle weakness and slow gait were both operationalized as performance-based measurements of handgrip strength and gait speed. Muscle weakness was defined as being at or below the 1st quintile of handgrip strength, adjusted by BMI and sex. BMI categories were used as cut-off points. Weight and height measurements were used to estimate BMI for CRELES participants; whereas weight and height self-reports were used for NHATS participants since no measurements were conducted in that cohort. Slow gait was defined as being at or above the 4th quintile of time in the walking-speed test, adjusted by height and sex. Median height was used as the cut-off point for each cohort. People who needed to use a cane or a walker to perform the walking-speed test in NHATS were classified as slow.

For both muscle weakness and slow gait components of the PFP index, we classified participants as weak and slow when participants did not attempt to conduct the test for safety concerns, or when they attempted but were unable to complete the test. Previous studies have made similar methodological decisions as these types of missing values ​​are likely to indicate poor performance and have been associated with mortality [18–20].

Participants were classified as *frail* if they met three or more of the PFP criteria, *pre-frail* if they met one or two criteria, and *non-frail* if they met none of the five criteria [8]. Cut-off points used for this study are included in Supplementary materials.

### Mortality assessment

The primary outcome was all-cause *mortality*. For Costa Rican participants, follow-up was accomplished by linking their personal unique identification numbers to the Costa Rican Death Index. For US participants, the follow-up process was dependent on interviews conducted to the proxy informants of deceased participants. Month and year of death was collected for both cohorts. Participants were followed up since their baseline interview. A sensitivity analysis, excluding deaths that occurred within 12 months, was also performed in order to assess if survival bias associated with frailty categories was introduced. To avoid bias from COVID-19 related deaths, and to have comparable cohorts in terms of follow-up length, mortality follow-up time was right censored at 8 years from the date at baseline interview. CRELES baseline interviews were conducted primarily in 2005, but the complete round of baseline interviews took place between 2004 and 2007. Therefore, censoring in CRELES occurred between December 2012 and December 2015. NHATS baseline interviews were conducted in 2011, therefore censoring occurred in December 2019, before the beginning of the COVID-19 pandemic in 2020.

### Covariates

In addition to frailty, we used the following self-reported variables at baseline to adjust for sociodemographic characteristics and health behaviors that are proximate determinants of mortality variation: *age, sex, education level, economic vulnerability, race and ethnicity, living in a metropolitan zone, marital status*, and *smoking*. Those variables that may have a causal relationship with frailty, such as chronic conditions, were not included in the mortality models. Similarly, those variables that are in the causal pathway between frailty and mortality, such as falls, hip fracture, and hospitalizations, were not included in order to avoid overcontrolling. We measured age by 5-yr age groups and used a continuous variable in the multivariate models. Sex had two categories: female or male. Education level was categorized as low, medium or high. For Costa Rican participants these three education levels respectively corresponded to: complete elementary school or lower, incomplete high school, and complete high school or higher. Because of the educational attainment of Costa Rican older adults, this is the typical approach to categorize education in the Costa Rican context. For US participants these three education levels corresponded to: incomplete high school or lower, complete high school or college education without obtaining a degree, and bachelor’s degree or higher.

Economic vulnerability was a dichotomous variable. Similar to previous studies, economic vulnerability was defined as a total income < 100 USD per month per person in 2014 USD for CRELES participants [21]. Also similar to previous studies, being a recipient of Medicaid was used as a proxy for economic vulnerability for NHATS participants, because this insurance is only available to older adults with low income and few assets [22]. Although race is known to be an important determinant of health outcomes in the US, it is not a health determinant in Costa Rica – indeed, race is not collected in Costa Rican population surveys. Therefore, only for the US model, race and ethnicity was used as a covariate and an interaction between frailty and race was introduced in order to assess whether race and frailty combinations had a modifying effect on mortality. We dichotomized race as White vs. all other race/ethnicity because of power issues when interacting with the frailty measurement. Living in a Metropolitan zone was also a dichotomous variable. Marital status was dichotomized as being married or in domestic partnership vs. not. Smoking behavior was dichotomized as being a current smoker at baseline.

### Statistical analysis

Characteristics of both populations at baseline are shown as relative frequencies (%). Differences in baseline characteristics between cohorts were tested using the χ2 test. Cox proportional hazards models were used to assess the association between frailty (measured in 3 categories), and all-cause mortality for each cohort. The appropriateness of the Cox models was checked by log-log plots. Models were fully adjusted, and survey weights were used. Analyses were performed using statistical software Stata 17 [23]. Statistical significance was set at p-value < 0.05.

## Results

There were 1,790 participants from CRELES and 6,680 participants from NHATS included in the analyses. Table 1 shows the baseline characteristics for participants. The mean age of CRELES respondents was 72.65 years (95%CI: 72.37–72.94), while the mean age of NHATS respondents was 74.55 (95%CI: 74.36–74.73). The distribution of frailty status was significantly different between countries (p < 0.001) Pre-frailty was more prevalent in Costa Rica (67.2% vs. 57.4%), whereas frailty was less prevalent in Costa Rica than in the US (16.2% vs. 21.9%). Compared to Costa Rican respondents, the US respondents were older (p < 0.001), had higher proportion female (p = 0.021), higher educational level (p < 0.001), lower economical vulnerability (p < 0.001), higher proportion living in a Metropolitan zone (p < 0.001), and higher prevalence of smoking (p < 0.001).

**Table 1:** Cohort characteristics at baseline, Costa Rica and the United States (weighted estimates)

	Costa Rica (n = 1790)	United States (n = 6680)	p-value
**Physical Frailty Phenotype (PFP)**			< 0.001
Non-frail	16.7	20.8	
Pre-frail	67.2	57.4	
Frail	16.2	21.9	
**Age**			< 0.001
65–69	38.8	30.1	
70–74	27.7	26.2	
75–79	18.8	19.4	
80–84	9.6	14.2	
85–89	3.8	7.4	
90+	1.4	2.7	
**Sex**			0.021
Male	47.6	44.2	
Female	52.4	55.8	
**Education level** ^**a**^			< 0.001
Low	50.3	24.3	
Medium	36.2	60.9	
High	13.5	14.8	
**Economic vulnerability** ^**b**^			< 0.001
Yes	37.1	10.5	
No	62.9	89.5	
**Race and ethnicity** ^**c**^			NA
White		81.1	
All other race/ethnicity		18.9	
**Metropolitan zone**			< 0.001
Metropolitan	55.0	81.6	
Non-Metropolitan	45.0	18.4	
**Marital status** ^**d**^			0.475
In union	58.6	59.6	
Not in union	41.4	40.4	
**Smoking** ^**e**^			< 0.001
Current smoker	9.0	53.5	
Non-smoker	91.0	46.5	

^**a**^ n = 6014 for NHATS. Low level of education was defined as elementary school or lower for Costa Rican participants, and incomplete high school or lower for the United States participants. Medium level of education was defined as incomplete high school for Costa Rican participants, and complete high school or college education without obtaining a degree for the United States participants. High level of education was defined as complete high school or higher for Costa Rican participants, and bachelor’s degree or higher for the United States participants

^**b**^ n = 1785 for CRELES and 6517 for NHATS. Economic vulnerability was defined as a total income < 100 USD per month per person in 2014 USD for CRELES participants, and as being a recipient of Medicaid for NHATS participants

^**c**^ n = 6289 for NHATS

^**d**^ n = 6674 for NHATS

^**e**^ n = 1786 for CRELES and 6675 for NHATS

As shown in Table 2, the US cohort had a lower prevalence of pre-frailty and a higher prevalence of frailty for every variable except high education level. For example, the prevalence of pre-frailty was lower, and the prevalence of frailty was higher for both males and females in the US cohort. In the US, the prevalence of frailty was lower among White than among individuals of other races or ethnicities (p < 0.001) (Table 2).

**Table 2:** Cohort characteristics at baseline by physical frailty status, Costa Rica and the United States (weighted estimates)

	Non frailty		Pre-frailty		Frailty		p-value^a^
	Costa Rica	United States	Costa Rica	United States	Costa Rica	United States	
**Sex**							
Male	19.9	25.9	65.7	55.7	14.5	18.5	< 0.001
Female	13.7	16.7	68.6	58.7	17.7	24.6	< 0.001
**Education level** ^**b**^							
Low	12.0	11.7	67.9	53.9	20.1	34.5	< 0.001
Medium	16.8	18.8	69.7	59.3	13.5	21.9	0.001
High	33.5	30.2	57.8	57.5	8.7	12.3	0.416
**Economic vulnerability** ^**c**^							
Yes	12.6	8.71	67.1	50.6	20.3	40.7	< 0.001
No	19.0	22.0	67.2	58.1	13.8	19.9	< 0.001
**Race and ethnicity**							< 0.001
White		22.0		57.7		20.3	
All other race/ethnicity		15.5		55.9		28.7	
**Metropolitan zone**							
Metropolitan	17.2	21.4	66.9	56.5	16.0	22.1	< 0.001
Non-Metropolitan	16.0	18.0	67.6	61.0	16.4	21.0	0.015
**Marital status**							
In union	19.7	25.0	67.2	57.7	13.0	17.3	< 0.001
Not in union	12.3	14.6	67.1	56.8	20.6	28.6	0.015
**Smoking**							
Current	13.8	20.7	70.8	57.3	15.4	21.9	< 0.001
Non-smoker	16.9	20.8	66.9	57.4	16.3	21.8	< 0.001

^a^ p-value compares the distribution of frailty categories, between Costa Rica and the United States, except for race and ethnicity that refers only to the United States

^b^ Low level of education was defined as elementary school or lower for Costa Rican participants, and incomplete high school or lower for the United States participants. Medium level of education was defined as incomplete high school for Costa Rican participants, and complete high school or college education without obtaining a degree for the United States participants. High level of education was defined as complete high school or higher for Costa Rican participants, and bachelor’s degree or higher for the United States participants

^**c**^ Economic vulnerability was defined as a total income < 100 USD per month per person in 2014 USD for CRELES participants, and as being a recipient of Medicaid for NHATS participants

At the end of 8 years of follow-up, 661 (34.5%) of individuals had died in the Costa Rican cohort and 1670 (26.3%) of individuals had died in the US cohort. Total person-years were 15,248 for CRELES and 30,928 for NHATS. Due to losses of follow-up, observational time was lower in the US. Losses of follow-up were not a concern in the first months of the study; median follow-up duration for censored individuals was 3.6 years in CRELES and 2.3 years in NHATS. Median follow-up duration was 8.2 years in CRELES, and 4.7 years in NHATS. Median follow-up duration among deceased and non-deceased individuals was 4.9 and 8.5 years in CRELES. Median follow-up duration among deceased and non-deceased individuals was 3.8 and 6.2 years in NHATS.

The prevalence of frailty at baseline was highest among those who died, followed by the prevalence among losses to follow-up, and lowest among non-deceased individuals: 28.6%, 16.1%, and 11.0% in Costa Rica (p < 0.001) vs. 41.2%, 20.7%, and 13.8% in US (p < 0.001).

After controlling for covariates, a significant dose effect of frailty on the hazard of all-cause death was observed, with pre-frail individuals having an increased hazard of death compared to non-frail older adults, and frail individuals having a higher hazard of death than pre-frail individuals.

**Fig. 1:**
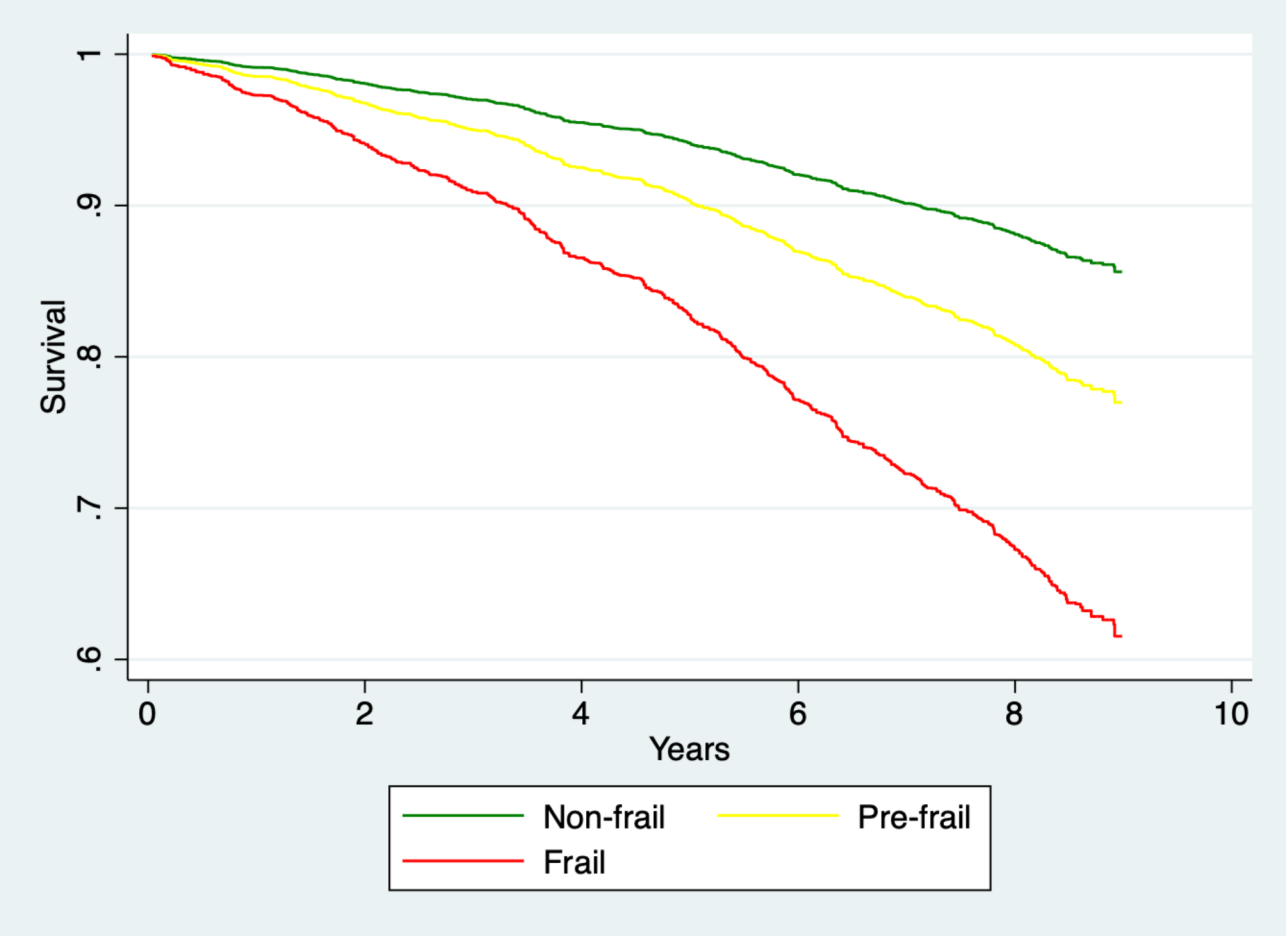
Costa Rica: Survival probability, by frailty status

**Fig. 2:**
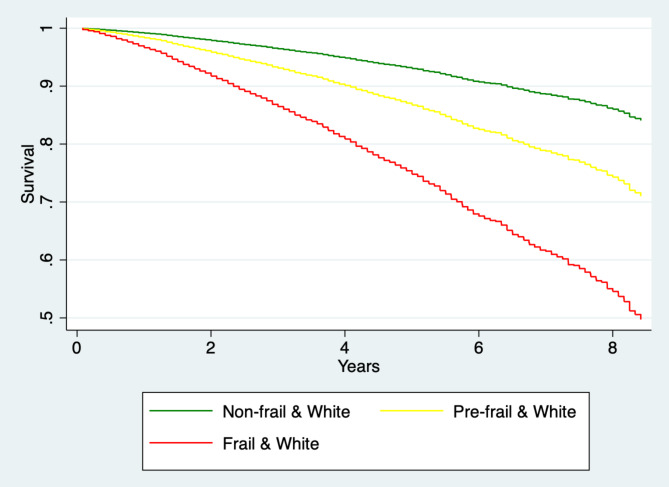
United States: Survival probability, by frailty status

The magnitude of the effect on pre-frail and frail individuals was higher in the US than in Costa Rica. Compared to non-frail individuals, pre-frail older adults had a 68% (p = 0.004) increased hazard of death in Costa Rica, and a 97% (p < 0.001) increased hazard of death in the US. The death hazard for frail as compared to non-frail older adults was three-fold in Costa Rica (HR = 3.13, p < 0.001) and four-fold in the White US (HR = 4.02, p < 0.001) (Figs. 1 and 2).

After performing a sensitivity analysis, in which deaths that occurred within 12 months from baseline were excluded to rule out survival bias, results remained similar. Compared to non-frail individuals, pre-frail older adults had increased hazards of death of 54% (p = 0.017) in Costa Rica, and 91% (p < 0.001) in the US. The death hazards for frail as compared to non-frail older adults were 2.86 (p < 0.001) in Costa Rica and 3.72 (p < 0.001) in the White US (Results not shown).

After controlling for other covariates, older age, being a male, and smoking were risk factors in both countries. Economic vulnerability was a risk factor in the US (HR = 1,31, p = 0,008). Having a high level of education was a protective factor in the US (HR = 0.60, p = 0.028), whereas being married or in a domestic union was a protective factor in Costa Rica (HR = 0.71, p = 0.001) (Table 3).

**Table 3:** Cox proportional Hazard Ratios from models evaluating the association between physical frailty and all-cause mortality. Costa Rica and the United States (weighted estimates)

	Costa Rica (n = 1790)			United States (n = 5936)		
	HR^a^	95% CI	p-value	HR^a^	95% CI	p-value
Pre-frail	1.68	1.19–2.39	0.004	1.97	1.52–2.55	< 0.001
Frail	3.13	2.12–4.61	p < 0.001	4.02	3.04–5.33	< 0.001
Age	1.07	1.05–1.08	p < 0.001	1.09	1.08–1.10	< 0.001
Men	1.51	1.23–1.84	p < 0.001	1.51	1.30–1.77	< 0.001
Medium education^**b**^	1.08	0.88–1.33	0.451	0.89	0.76–1.04	0.135
High education^**c**^	0.97	0.66–1.41	0.856	0.76	0.60–0.97	0.028
Economic vulnerability^**d**^	0.97	0.79–1.18	0.754	1.31	1.08–1.60	0.008
All other race/ethnicity				1.65	0.81–3.36	0.162
Pre-frail * all other race				0.48	0.23–1.00	0.051
Frail * all other race				0.41	0.17–0.95	0.039
Metropolitan zone	1.07	0.88–1.30	0.519	0.93	0.81–1.07	0.286
In union	0.71	0.58–0.87	0.001	0.89	0.79–1.00	0.058
Current smoker	1.51	0.11–2.06	0.008	1.40	1.22–1.60	< 0.001

^**a**^ Hazard Ratios (HR) estimations are fully adjusted (i.e., adjusted for frailty, age, sex, education level, economic vulnerability, living in a metropolitan zone, marital status, and smoking). US model is also adjusted for race & ethnicity and for interactions between race and frailty

^**b**^ Medium level of education was defined as incomplete high school for Costa Rican participants, and complete high school or college education without obtaining a degree for the United States participants

^**c**^ High level of education was defined as complete high school or higher for Costa Rican participants, and bachelor’s degree or higher for the United States participants

^**d**^ Economic vulnerability was defined as a total income < 100 USD per month per person in 2014 USD for CRELES participants, and as being a recipient of Medicaid for NHATS participants

**Table 4:** Cox proportional Hazard Ratios for interactions between frailty and race, from a model evaluating the association between physical frailty and all-cause mortality. United States (weighted estimates)

	HR^a^	95% CI	p-value^b^
Non-frail & White (Reference)			
Pre-frail & White	1.97	1.52–2.55	< 0.001
Frail & White	4.02	3.04–5.33	< 0.001
Non-frail & Other race/ethnicity	1.65	0.81–3.36	0.162
Pre-frail & Other race/ethnicity	1.56	1.18–2.08	0.003
Frail & Other race/ethnicity	2.70	2.00–3.66	< 0.001

^**a**^ Hazard Ratios (HR) estimations are fully adjusted (i.e., adjusted for frailty, age, sex, education level, economic vulnerability, race and ethnicity, interactions between race and frailty, living in a metropolitan zone, marital status, and smoking)

^**b**^ p-values are testing the null hypothesis of no interaction effect between race and frailty combinations, on mortality

**Fig. 3:**
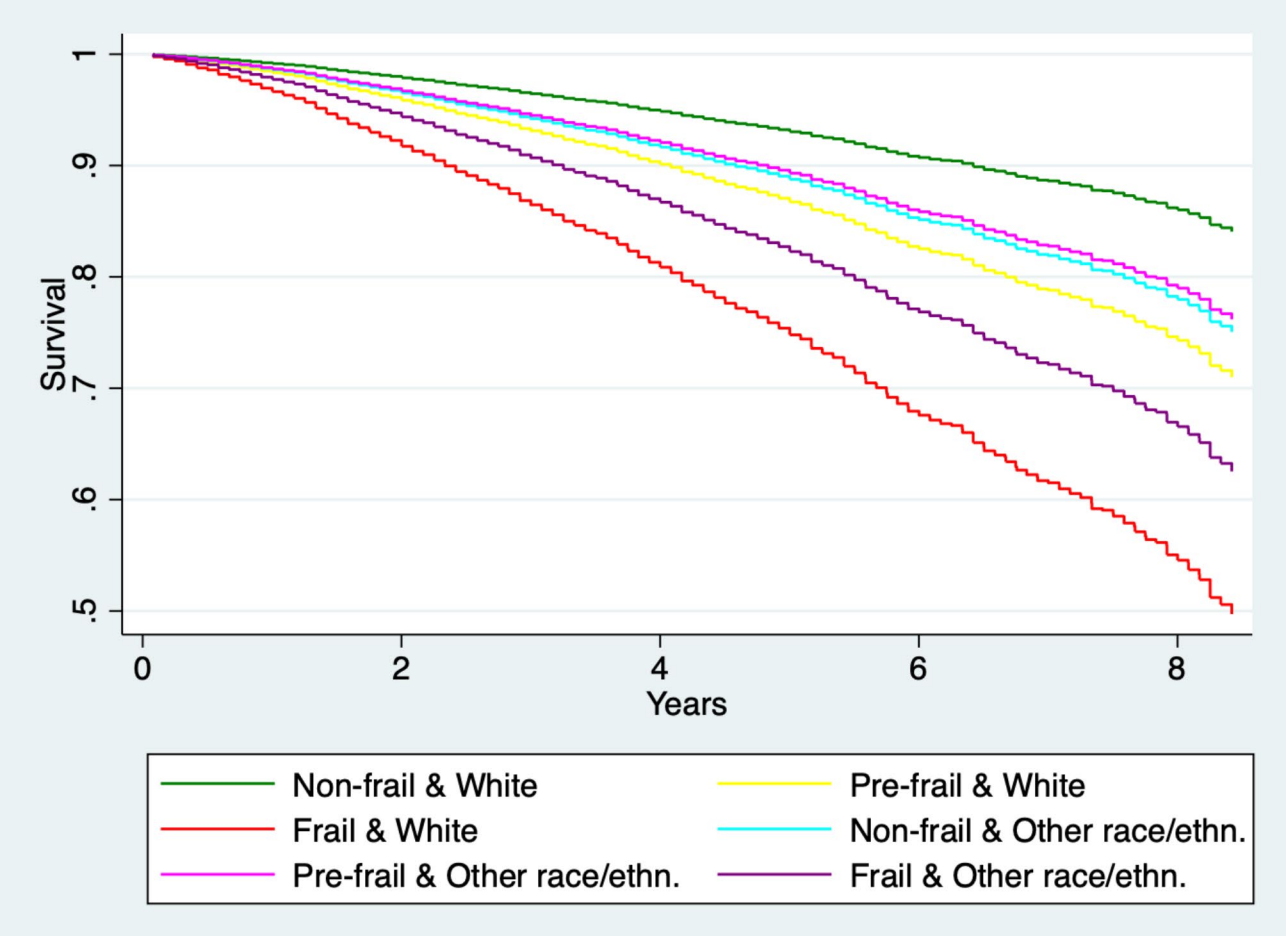
United States: Survival probability, by frailty and race interactions

Hazard ratios of death are greater for frail White individuals than for frail individuals of other races or ethnicities (HR = 4.02, p < 0.001 vs. HR 2.70, p < 0.001) and this disadvantage holds for pre-frail White individuals compared to pre-frail individuals of other races or ethnicities (HR = 1.97, p < 0.001 vs. HR 1.56, p = 0.003) (Table 4; Fig. 3).

## Discussion

Using nationally representative data from community-dwelling older adults, this study examined the association between frailty and mortality in Costa Rica and the United States. Adding to the limited existing literature comparing the experience of aging between these two countries, one developing and one developed, we found some support for our hypothesis that frailty can have a differential impact on mortality depending on the country, which may be due to differences in the social and economic contexts. Specifically, our results indicate that US older adults have a greater frailty prevalence, and that frailty has a stronger association with mortality than in Costa Rica. Indeed, our analyses suggest several differences between these two countries, which we discuss in more detail here.

We used the Physical Frailty Phenotype to assess the frailty status of two nationally representative cohorts of older adults from Costa Rica and the US. In Costa Rica, the prevalence of frailty (16.2%) was below the prevalence estimated by a systematic review and meta-analysis in the Latin American and the Caribbean Region using different frailty measurement approaches (19.6%) [24]. Costa Rican prevalence was nonetheless greater than the estimation of frailty prevalence of 12.7% from a systematic review and meta-analysis of studies using the PFP approach involving community-dwelling older adults ages 60 and older from upper middle-income countries in Latin America and the Caribbean [3].

In the United States, the prevalence of frailty (21.9%) was higher than estimated by a previous study (15.0%) [25]. Differences between these two estimations might be due to methodological differences, such as our exclusion of respondents living in residential care settings or some differences in the measurement of frailty. For example, differences in walking speed and grip strength cut-off points, which result from the percentile distribution of individuals in each analytical sample, can be contrasted from both studies’ supplementary tables. A systematic review conducted with 21 cohorts of community-dwelling older adults ages 65 and over estimated a lower prevalence of frailty in high income countries when only studies using the PFP were included (13.6%) [2]. In European countries the prevalence of frailty ranges from 5.8% in Switzerland to 27.3% in Spain [26]. However, the latter estimates were based on a related but not equivalent measurement of Fried’s frailty, which attempted to operationalize PFP with the available data [6].

Similar to the current study, characteristics such as being female, older age, and being economically vulnerable have previously been found to be associated with the prevalence of frailty in the US [25]. Research studies in different contexts have consistently found a higher prevalence of frailty in the female older adult population. This gender differential has been described as a gender-approach to frailty [6]. A sex-frailty paradox has also been described, with women having greater levels of frailty but living longer than men [27, 28], which holds for both cohorts included in this study. Interestingly, this sex-frailty paradox has not been observed in other species – for example, mice have been found to have an increased prevalence of frailty as they age, with frail mice dying earlier, regardless of sex [29]. The sex-frailty paradox has not been supported by data in primate populations of baboons either, with similar age-related health outcomes, regardless of sex [30]. Cultural norms and behaviors may be associated with the sex differential observed in human populations as long as they imply environmental non-biological differences with an effect on health outcomes [31]. Roles that have been culturally assigned to women, such as caregiving, may increase the risk of frailty [32], while behaviors that have been culturally modeled in men, such as a lower utilization of healthcare services [33], may increase mortality among frail males.

Similar to our results, previous studies have also found an increased mortality risk in frail older adults. Another study using NHATS found that death was significantly higher among frail compared to non-frail participants [34]. A Chinese cohort study reported that using the frailty index, the hazard ratio of all-cause mortality risk was higher in frail older adults [35]. Similarly, a meta-analysis found that frailty status measured with the PFP was associated with an increased risk of all-cause mortality in community-dwelling older adults [11]. We found that besides frailty, increasing age, being male, and smoking are risk factors for death in both countries. A high education level in the US and being married/in union in Costa Rica were found to be protective factors according to our study. Similar results have been identified in previous investigations [35, 36].

The highest prevalence of pre-frailty was found in Costa Rica, and the highest prevalence of frailty was found in the US. Despite having better indicators in terms of education and socioeconomic status, the US older adults had a greater frailty prevalence and the magnitude of its association with mortality was also higher than in Costa Rica. Previous studies have shown that socioeconomic inequality in mortality is greater in the US than in Costa Rica, with individuals on the lowest socioeconomic status in Costa Rica living longer than individuals in a similar social position in the US [37]. Having health insurance at older ages would not explain these differences, because all the participants in the US cohort are Medicare recipients, and all of the participants in the Costa Rican cohort are also entitled to health insurance. However, one explanation for these country differences is access to a universal health care system across the life cycle in Costa Rica which offers an excellent primary care access and provides a safety net for individuals regardless of their socioeconomic status. Features of Costa Rica’s health care system may slow the progression from non-frail to pre-frail and to frail, which may explain the differences observed at the end-of-life trajectories. In the US, public health insurance under the age of 65 is primarily available to those with very low incomes, while the majority access private health insurance through their employers or out of pocket. In contrast, in Costa Rica almost all residents are covered by a single national health insurance system [29]. In addition, Costa Rica’s higher life expectancy when compared to other developing countries has been attributed in part to the availability of primary care, whereas primary care accounts for less than 7% of total health expenditures in the US [38].

Another explanation besides differences in health care systems relates to psychosocial pathways that involve stress levels, anxiety, social isolation, and depression. The mortality risk from loneliness and social isolation is well established; results from a meta-analysis of 70 studies suggest the risk of death for those without social connections and support is comparable to obesity, lack of physical activity, limited access to health care, and similar risk factors [39]. Shorter telomeres in blood cells indicate that US residents may live under more stressful circumstances than Costa Ricans [37].

As expected, this study found that White older adults had a significantly lower prevalence of frailty compared to individuals of other races or ethnicities in the US. This result is consistent with studies that have previously found racial and ethnic disparities in the US, with Black and Latino older adults showing a higher prevalence of frailty [25, 40]. However, a surprising result was that although White individuals had a lower prevalence of frailty, those who were pre-frail or frail faced a higher hazard of death compared to other races or ethnicities. Although Black and Latino populations show higher mortality in most age groups, a mortality crossover with these populations having a comparable or lower mortality than Whites has been previously described for older adults in the US [41]. A possible explanation for this observed mortality advantage of other races and ethnicities is differential selective survival [42]. Using the same NHATS survey, a previous study reported a modestly lower mortality risk for Black older adults compared to White individuals [43]. Black older adults may have greater availability of informal networks of support within their families and communities [43], suggesting the need for future research to explore the effects of informal support on racial differentials in health outcomes.

The strengths of this study include a prospective design with nationally representative samples of older adults that provides evidence of mortality risk from two different country contexts. Moreover, we used a reliable frailty indicator for community-based population samples that allowed for cross-country comparisons.

This study also has limitations. In both cohorts, selection bias may result from the fact that losses to follow-up had a higher prevalence of frailty than those who were not lost, which would result in a lower estimation of the association between frailty on mortality. Residual confounding may result from chronic conditions associated with frailty and mortality which were not included in the analyses. Recall bias may come from self-reports of three out of the five PFP components which were not based on performance tasks. We adapted the definitions of weight loss and physical activity of the PFP criteria to make them comparable using the available data with measurements made in the CRELES and NHATS cohorts, however they differ from the study on which the phenotype is based [8]. Furthermore, the PFP does not allow us to assess the severity of frailty, and there are likely variations for older adults in this category. As a result of the methodological differences in mortality follow-up between countries, completeness and accuracy of the mortality outcome are expected to be higher in Costa Rica, where a linkage to the official Death Index was used, as compared to the US where the information relied on proxy reports. This measurement bias may lead to mortality sub estimation in the US population, which would result in greater differences between countries. Country differences may also result from differential paces of aging that were defined by biological and environmental factors earlier in life [44, 45]. A limitation of this study is the lack of longitudinal measures during adulthood to assess whether the pace of aging differed by country.

## Conclusions

The death hazard for frail compared to non-frail older adults was three-fold in Costa Rica and four-fold in the US. Older age, being male, and smoking were death risk factors in both countries. High education was a protective factor in the US, whereas being married or in union was a protective factor in Costa Rica. While our study did not explicitly examine the reasons for differences in frailty prevalence and its association with mortality, prior work exploring how the surrounding context affects the health and well-being of residents of these two countries suggest some possible explanations, including access to healthcare at earlier ages, social connection and support, and the role of racism as a social determinant of health in the US. Future research should seek to identify behavioral, social, and policy determinants of frailty that may serve as an essential component of population interventions that prevent frailty and its consequences.

### Electronic supplementary material

Below is the link to the electronic supplementary material.

Supplementary Material 1

## Data Availability

The datasets used in this study can be found in online repositories at: http://www.creles.berkeley.edu/ and https://nhats.org/ for CRELES and NHATS respectively.

## List of abbreviations

CRELES: Costa Rican Longevity and Healthy Aging Study, for its Spanish acronym
NHATS: National Health & Aging Trends Study
PFP: Physical Frailty Phenotype
US: United States of America
