# Supplementary material for: Frailty as a predictor of mortality: a comparative cohort study of older adults in Costa Rica and the United States
Source: BMC Public Health. 2023 Oct 10;23:1960. doi: 10.1186/s12889-023-16900-4 (PMC10563325; doi:10.1186/s12889-023-16900-4)
Supplement: Supplementary file 1 — Supplementary Material 1 [file 12889_2023_16900_MOESM1_ESM.docx]

**SUPPLEMENTARY MATERIALS**

**Supplementary Table 1.** Quintile 1 of walking speed by sex and height, Costa Rica.

| **Sex, height** | **Speed (m/s)** | **n** |
| --- | --- | --- |
| Female, <= 149.6 cm | 0.404 | 97 |
| Female, > 149.6 cm | 0.429 | 103 |
| Male, <= 163.2 cm | 0.456 | 89 |
| Male, > 163.2 cm | 0.492 | 86 |
| Tried but unable, not attempted for safety or incapacitated | | 146 |

Note. Within sex-by-median-height categories; persons below quintiles displayed meet frailty criterion for “slowness”; n is the number of participants (by category) meeting this criterion.

**Supplementary Table 2.** Quintile 1 of walking speed by sex and height, United States.

| **Sex, height** | **Speed (m/s)** | **n** |
| --- | --- | --- |
| Female, <= 160.05 cm | 0.500 | 229 |
| Female, > 160.05 cm | 0.545 | 243 |
| Male, <= 175.35 cm | 0.600 | 223 |
| Male, > 175.35 cm | 0.667 | 223 |
| Tried but unable, not attempted for safety | | 399 |
| Incapacitated | | 359 |

Note. Within sex-by-median-height categories; persons below quintiles displayed meet frailty criterion for “slowness”; n is the number of participants (by category) meeting this criterion.

**Supplementary Table 3.** Quintile 1 of grip strength by sex and BMI, Costa Rica.

| **Sex, BMI** | **Strength (kg)** | **n** |
| --- | --- | --- |
| Female, Underweight: BMI < 18.5 | 9.65 | 6 |
| Female, Normal: BMI 18.5 - <25 | 14.00 | 58 |
| Female, Overweight: BMI 25 - <30 | 15.75 | 81 |
| Female, Obese: BMI >= 30 | 15.50 | 67 |
| Male, Underweight: BMI < 18.5 | 21.65 | 4 |
| Male, Normal: BMI 18.5 - <25 | 22.70 | 65 |
| Male, Overweight: BMI 25 - <30 | 26.00 | 88 |
| Male, Obese: BMI >= 30 | 22.25 | 32 |
| Tried but unable, not attempted for safety or incapacitated | | 31 |

Note. Within sex-by-BMI categories; persons below quintiles displayed meet frailty criterion for “weakness”; n is the number of participants (by category) meeting this criterion.

**Supplementary Table 4.** Quintile 1 of grip strength by sex and BMI, United States.

| **Sex, BMI** | **Strength (kg)** | **n** |
| --- | --- | --- |
| Female, Underweight: BMI < 18.5 | 13.26 | 16 |
| Female, Normal: BMI 18.5 - <25 | 14.45 | 192 |
| Female, Overweight: BMI 25 - <30 | 15.03 | 207 |
| Female, Obese: BMI >= 30 | 15.10 | 197 |
| Male, Underweight: BMI < 18.5 | 23.69 | 5 |
| Male, Normal: BMI 18.5 - <25 | 23.05 | 132 |
| Male, Overweight: BMI 25 - <30 | 26.05 | 218 |
| Male, Obese: BMI >= 30 | 26.11 | 142 |
| Tried but unable, not attempted for safety or incapacitated | | 57 |

Note. Within sex-by-BMI categories; persons below quintiles displayed meet frailty criterion for “weakness”; n is the number of participants (by category) meeting this criterion.
